# Assessment of BoAHV-1 Seronegative Latent Carrier by the Administration of Two Infectious Bovine Rhinotracheitis Live Marker Vaccines in Calves

**DOI:** 10.3390/vaccines12020161

**Published:** 2024-02-03

**Authors:** Stefano Petrini, Cecilia Righi, Giulia Costantino, Eleonora Scoccia, Paola Gobbi, Claudia Pellegrini, Michela Pela, Monica Giammarioli, Giulio Viola, Roberto Sabato, Elena Tinelli, Francesco Feliziani

**Affiliations:** 1 National Reference Centre for Infectious Bovine Rhinotracheitis (IBR), Istituto Zooprofilattico Sperimentale Umbria-Marche, “Togo Rosati”, 06126 Perugia, Italy; c.righi@izsum.it (C.R.); g.costantino@izsum.it (G.C.); e.scoccia@izsum.it (E.S.); p.gobbi@izsum.it (P.G.); c.pellegrini@izsum.it (C.P.); m.pela@izsum.it (M.P.); m.giammarioli@izsum.it (M.G.); r.sabato@izsum.it (R.S.); e.tinelli@izsum.it (E.T.); f.feliziani@izsum.it (F.F.); 2 Viola Giulio dairy cattle farm, 62026 Macerata, Italy; violagiulio@gmail.com

**Keywords:** calves, IBR, SNLC, live marker vaccines

## Abstract

Seronegative latent carriers (SNLCs) are animals that carry the virus without detectable antibodies and pose a risk for disease transmission and diagnostic challenges, suggesting the importance of consideration of marker vaccines in managing them. Therefore, in this study, we evaluated two modified live infectious bovine rhinotracheitis (IBR) marker vaccines (single and double deletions) for their ability to generate SNLC calves. These vaccines were administered to four groups (n = 3 in each group) of three-month-old calves in the presence or absence of passive immunity. Three hundred days after the first vaccination and after confirming the IBR seronegativity of all animals, dexamethasone was administered intravenously for five consecutive days. Only animals immunized with the modified live IBR marker vaccine (single deletion) in the absence of passive immunity exhibited a more enduring immune response than those vaccinated in the presence of passive immunity. Moreover, the administration of a modified live IBR marker vaccine (double deletion) to calves with passive immunity generated SNLC. These findings underscore the potential of live IBR marker vaccine (double-deletions) to aid serological diagnostic tools and develop vaccination protocols in achieving the desired immune response, particularly in the context of latent carrier status, offering valuable insights into optimizing vaccination strategies for effective IBR control.

## 1. Introduction

Bovine alphaherpesvirus 1 (BoAHV-1) is a member of the *Alphaherpesviridae* subfamily and genus *Varicellovirus* [1]. This etiological agent is associated with infectious bovine rhinotracheitis (IBR) disease and several clinical syndromes, including infectious pustular vulvovaginitis (IPV) and infectious balanoposthitis (IBP). In addition, the virus has been implicated in several afflictions, such as conjunctivitis, encephalitis, endometritis, infertility, abortions, and enteritis [2]. BoAHV-1 is causing significant economic loss all over the world.

In recent years, many European Union (EU) countries have initiated a consensus IBR eradication program [3]. In March 2016, the European Parliament and European Council adopted Regulation (EU) 2016/429 for transmissible animal diseases (“Animal Health Law”). These regulations were implemented since 21 April 2021. The successive Regulation (EU) 2018/1882, published in December 2018, categorizes each of the listed diseases, including IBR, which has been listed in the C+D+E category [4,5].

European programmes to control and eradicate IBR have been hampered by the use of conventional vaccines. In contrast, in the USA, only modified live or inactivated vaccines are used. Regarding safety and efficacy, these products have various disadvantages. Therefore, new vaccination strategies against BoAHV-1 have focused on designing IBR marker vaccines. These products have the deletion of the glycoprotein E (gE-) of BoAHV-1 or the deletion of both gE and thymidine-kinase enzyme (gE-/tk-) of BoAHV-1. The gE deletion of BoAHV-1 allowed the development of an ELISA test capable of discriminating immunized animals with the IBR marker vaccine from infected cattle. This strategy is known as Differentiating Infected from Vaccinated Animals (DIVA) [3].

Currently, it is possible to identify latently infected animals by detecting circulating antibodies; the presence of passive immunity against BoAHV-1 can potentially impede the immune response following infection [6] or vaccination [7]. Based on this, the European study group studying IBR—as part of the “DISease CONtrol TOOLS” (DISCONTOOLS)—identified a crucial *gap* in the main perceived obstacles to effective prevention and control of IBR. However, evidence from IBR virus/IBP outbreaks in two UK bull studs indicated that seronegative latent carrier (SNLC) calves represent a significant source of infection. This highlighted that the policy of procuring young bulls with maternal antibodies against BoAHV-1 presents an unquantified risk of introducing SNLC calves into the study [8].

Lemaire et al. [9] demonstrated the feasibility of generating an SNLC through the immunization of animals with passive immunity using a live attenuated temperature-sensitive (*ts*) vaccine. In addition, several authors have demonstrated the absence of seroconversion after vaccination with *ts* in animals with passive immunity and a latent state has never been observed. This indicates the urgency of assessing modified-live IBR marker vaccines (single and double deletions) to generate SNLC.

Therefore, in this study, we hypothesized that administering modified-live IBR marker vaccines (single and double deletions) to calves with passive immunity can generate SNLC animals. To test this hypothesis, we aimed to investigate whether vaccination of calves with passive immunity (transferred from dams previously immunized with IBR marker inactivated vaccines) using single and double deletion live IBR marker vaccines could generate SNLC calves.

## 2. Materials and Methods

### 2.1. Experimental Designs

Twelve Italian Friesian calves of approximately 3 months of age selected from a dairy herd in Central Italy (Marche Region) were used in this study. Farm records showed no recent history of respiratory diseases. The calves were divided into four groups (groups 1–4), each comprising three calves (Table 1). The animals in groups 1 and 3 were fed IBR-free commercial pasteurized bovine colostrum (Immune Milk, Phytobiotics Futterzusatzstoffe GmbH, Eltville, Germany). Briefly, 500 g freeze-dried colostrum containing 20% fat, 47% crude protein, and 38% immunoglobulins was dissolved in 2 L water and heated to 37 °C. The solution was fed to calves twice on the first and second days of birth. Subsequently, the calves were fed milk collected from IBR-free cattle until weaning (at 3 months of age). The calves in groups 2 and 4 were fed colostrum and milk collected from cattle previously immunized (at five months of gestation) with an inactivated glycoprotein E-deletion marker vaccine. Milk was administered until weaning (at 3 months of age).

The care and experimental protocols were in accordance with the Directive 2010/63/EU of the European Parliament and the Council on the protection of animals used for scientific purposes [10]. Furthermore, the experiments were authorized (no. 423/2020-PR and no. 618/2022-PR) by the Italian Ministry of Health.

**Table 1:** Modified live IBR marker vaccines used in the experiment.

Group	No. of Calves	Age (Months)	IBR Passive Immunity	Vaccine Identification	Type (Deletion)	Virus Concentration (TCID_50_/mL) ^c^	Inoculation Route
1	3	3	Negative ^a^	A	gE^−^	10^6.24^	Intranasal
2	3	3	Positive ^b^	A	gE^−^	10^6.24^	Intranasal
3	3	3	Negative ^a^	B	gE^−^/tk^−^	10^5.74^	Intramuscular
4	3	3	Positive ^b^	B	gE^−^/tk^−^	10^5.74^	Intramuscular

^a^ The calves were fed with colostrum “Immune Milk IBR free” (Phytobiotics Futterzusatzstoffe GmbH) on days 1 and 2 after their birth, followed by milk collected from IBR-free cattle until weaning (3 months of age). ^b^ The calves were fed with colostrum and milk collected from previously immunized cattle (at five-month of gestation) with inactivated gE^−^deletion marker vaccine; ^c^ TCID_50_/mL determined by Reed and Muench Method [11] using Madin–Darby Bovine Kidney (MDBK) cell culture. The milk was administered until weaning (3 months of age). gE, glycoprotein E of BoAHV-1; tk, thymidine-kinase enzyme of BoAHV-1; TCID_50_/mL, Median Tissue Culture Infectious Dose.

The number of animals per group was calculated using a 5% margin of error and a power of 80% for a clinical trial comparing superiority ratios. In the control and experimental groups, 0% and 90%, were considered for the proportion of events occurring.

The calves in the four groups were employed in this study for different sets of experiments, as described in the following sections.

### 2.2. Immunization

Two modified-live IBR marker vaccines (A, single-deletion; B, double-deletion) were used in this study (Table 1). Two doses (2 mL each) of these vaccines were administered to each animal, 21 days apart, starting at the age of 3 months, using the concentrations and inoculation routes shown in Table 1.

Calves in groups 1 and 2 were immunized with vaccines A (a gE^−^deleted IBR live marker vaccine) and those in groups 3 and 4 with vaccine B (a gE^−^/thymidine-kinase (tk)^−^deleted IBR live marker vaccine). Each animal was initially inoculated with a 2 mL dose of vaccine, and after three weeks, a booster (2 mL dose) was administered. Calves were monitored until BoAHV-1 seronegativity was observed (at approximately 10–12 months of age).

After the vaccination, the animals were observed clinically for 30 days. The clinical parameters considered were (i) nasal discharge, (ii) eye discharge, (iii) ear score, (iv) cough score, (v) rectal temperature score, (vi) fecal score, and (vii) joint score. The assigned scores range from 0 (normal clinical signs) to 3 (severe clinical signs).

At 0, 15, 21, 28, 60, 90, 120, 180, 210, 240, 270, and 300 days post-vaccination (DPV), nasal swabs and blood samples were collected for virological investigation (virus isolation and gB-specific real-time PCR). Additionally, serum samples were collected at the time points mentioned above for serological investigations (ELISA and Virus Neutralization test).

### 2.3. Vaccine Reactivation

To demonstrate the vaccine reactivation, at seronegativity to BoAHV-1 (around 10–12 months of age), the animals were treated with dexamethasone (DMS; Dexadreson^®^, MSD Animal Health S.r.l., Milan, Italy). Each calf received an intravenous injection of 0.1 mg DMS/kg body weight for 5 consecutive days. The calves were then clinically observed for 30 days, and the clinical parameters considered were the same as those reported in Section 2.1. At 0, 2, 3, 4, 7, 10, 14, and 21 days post-treatment with DMS (DPT-DMS), nasal swabs and blood samples were collected for virological investigation (virus isolation and gB-specific real-time PCR) and serum samples were collected for serological investigations (ELISA and Virus Neutralization test).

### 2.4. Samples Collection

Each nasal swab was collected by rubbing both nasal cavities five times and was then immersed in a test tube (Citoswab^®^, Wlkang LTD, London, UK) containing 3 mL minimal essential medium (MEM; Euroclone, Milan, Italy) plus 5 × antibiotics (5000 I.U. Penicillin, 2550 I.U. Streptomycin, and 25 µg Amphotericin B; Euroclone, Milan, Italy).

Blood samples (approximately 8 mL from each calf) were collected from the coccygeal or jugular veins using disposable and sterile vacutainers (Vacutest^®^, Vacutest Kima s.r.l., Padua, Italy) and needles 20 G (Vacuette^®^ Multiple Use Drawing Needle, Greiner Bio-One Gmbh, Kremsmunster, Austria). Subsequently, the blood samples were centrifuged at 850× *g* for 30 min at 4 °C to extract the serum for serological investigation.

The samples were transferred to the laboratory at 4 °C within 2 h from collection. Before testing the nasal swabs, the samples were stored at −80 °C. Blood and serum samples were refrigerated and used within two days.

### 2.5. Virus Isolation

Approximately 0.1 mL of each nasal swab was plated into three wells of a 24-well plastic plate (CytoOne^®^ Plate; Starlab LTD, Blakelands, UK) containing monolayers of MDBK cell cultures grown in MEM. The cells were identified as BS CL 63 and provided by the Biobank of Veterinary Resources (BVR; Brescia, Italy). Subsequently, 1 mL MEM supplemented with 2% fetal calf serum (BioWhittaker Inc., Walkersville, MD, USA) was added to each well after incubation for 60 min at 37 °C in a 5% CO_2_ atmosphere. MDBK cells infected with BoAHV-1 (Los Angeles reference strain; batch IZSUM No. 01/17) were used as positive controls. MDBK cell cultures free of BoAHV-1 were used as negative controls. Plates were incubated for 7 days at 37 °C in a 5% CO_2_ atmosphere and were observed daily to determine if a cytopathic effect (CPE) occurred. Viral titres were determined by the Reed and Muench method [11] and were expressed as mean tissue culture infectious doses (TCID_50_/mL). A direct immunofluorescence assay using an anti-BoAHV-1 monoclonal antibody (Bio026; Bio-X Diagnostic S.A., Rochefort, Belgium) identified the virus recovered from the samples as BoAHV-1.

### 2.6. ELISA Tests

Sera were tested using two commercially available ELISAs (IDEXX IBR gBX3 Ab and IDEXX IBR gE Ab; Westbrook, ME, USA), and results were interpreted according to the manufacturer’s instructions. An automatic plate reader (SunriseTM; Tecan AG, Männedorf, Switzerland) was used to read the plates, and Magellan software version 7.1 (Tecan, Mannedorf, Switzerland) was used to analyze the data.

### 2.7. Virus Neutralization Test (VNT)

Serum samples collected at different time points (see Section 2.2 and Section 2.3) were tested in parallel with BoAHV-1 positive, doubt, and negative reference serum samples. The VNT was performed according to a protocol described by the World Organization for Animal Health [12]. Briefly, 50 µL 100 TCID_50_ BoAHV-1 (Los Angeles reference strain; batch IZSUM No. 01/17) was added to 50 µL undiluted serum samples and twofold dilutions of each serum sample (from 1:2 to 1:2048). Each mixture was then transferred into 96-well microtiter plates (CytoOne^®^ Plate; Starlab LTD, Blakelands, UK) in triplicate. The plates, were incubated at 37 °C for 24 h. Subsequently, 100 µL of MEM containing 3 × 10^4^ MDBK cell cultures, plus 10% of fetal calf serum (Euroclone, Milan, Italy) were added to each well and incubated at 37 °C—5% CO_2_ for four days. The cells were identified using the BS CL 63 code and obtained from BVR. After incubation, the plates were examined using an inverted tissue culture microscope (Olympus IX51; Olympus Corporation, Tokyo, Japan) to determine the cytopathic effects. The neutralizing titer was expressed as the highest dilution that inhibited the cytopathic effect.

### 2.8. gB-Specific Real-Time PCR

A High Pure PCR Template Preparation Kit (Roche Diagnostics Deutschland, Mannheim, Germany), was used to extract viral DNA from nasal swabs according to the manufacturer’s instructions. gB-specific real-time PCR, was carried out using the World Organisation for Animal Health protocol [12] including an internal amplification control. The following modifications, were made to the probe: 50-VIC-TCG CTG TCC ACC TTC CAG CAG ATG T-TAMRA-3. All samples, were tested in duplicate and a sample was considered positive if the Ct value was equal to or less than 45.

### 2.9. Statistical Analysis

Neutralizing antibody (NA) titres were measured as log_10_. For each group of animals at all sampling times, the mean titers were calculated. Statistically significant differences between animals with and without passive immunity for each vaccine were evaluated using the Wilcoxon Mann-Whitney non-parametric test. Differences were considered statistically significant at *p* < 0.05. The analysis was performed using Stata software version 16.1 (StataCorp LCC, College Station, TX, USA).

## 3. Results

### 3.1. Clinical Response

**Figure 1:**
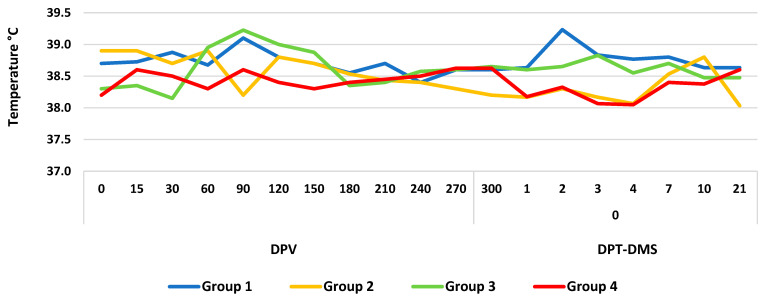
Rectal temperatures recorded in different groups immunized with a gE-deleted IBR live marker vaccine (groups 1 and 2) and a gE-/tk-deleted IBR live marker vaccine (groups 3 and 4) and treated with dexamethasone (DMS). DPV, days post-vaccination; DPT-DMS, days post-treatment with dexamethasone.

The tested vaccines did not cause any adverse reactions in any of the animals. However, groups 1 and 3 developed coughing, dyspnea, and nasal discharge on PVD 15. After the diagnosis of *Pasteurella Multocida*, the calves were treated with antibiotics (Oxytetracycline 20% Chemifarma, Forlì, Italy) for 7 days. In particular, 30 mg of the active principle per kilogram of body weight was administered to milk. At the end of the treatment period, all animals were cured. In addition, during the entire experimental period, none of the groups developed fever, and the rectal temperature ranged from 38.0 °C to 39.2 °C (Figure 1).

**Figure 2:**
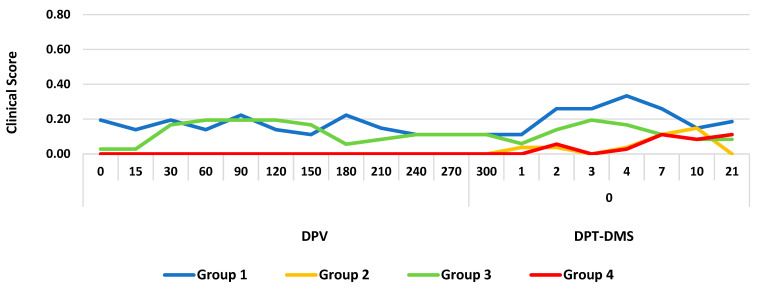
Clinical scores detected in different groups immunized with a gE-deleted IBR live marker vaccine (groups 1 and 2) and a gE-/tk-deleted IBR live marker vaccine (groups 3 and 4) and treated with dexamethasone (DMS); DPV, days post-vaccination; DPT-DMS, days post-treatment with dexamethasone.

Furthermore, during the vaccination period, the clinical scores of all the groups remained below 0.2. After treatment with DMS, a slight increase to 0.33 in the clinical score was observed only in group 3 on DPT-DMS 4 (Figure 2).

### 3.2. Serological Investigations

The NA titer was detected in group 1 on DPV 15 with an average titer of 0.401 log_10_. In contrast, group 2 showed an average NA titer of 2.308 log_10_ (*p* = 0.0039) on the day of the first vaccination (DPV 0), resulting from the transfer of colostral passive immunity. The NA concentration in group 1 progressively increased to 2.709 log_10_ (*p* = 0.0431) on DPV 90. Subsequently, the titer decreased up to 0.401 log_10_ (*p* = 0.0369) on DPV 240. In contrast, in group 2, the first vaccination intervention progressively reduced the mean NA titer to 0.702 log_10_ (*p* = 0.0463) on DPV 150 (Table 2). Concerning the results obtained with the gB-ELISA test, group 1 showed positivity on DPV 15, and the same was detected up to DPV 240. In contrast, group 2 was positive in the gB-ELISA test on DPV 0 owing to the presence of colostral passive immunity. Positivity was detected up to DPV 150. In addition, all animals showed negative results in the gE-ELISA test (Table 2).

In group 3, NA was first detected on DPV 15, with a mean titer of 0.903 log_10_ (*p* = 0.6579). They progressively increased, with a mean titer of 2.208 log_10_ (*p* = 0.0463) DPV 120, and then decreased, with a mean titer of 0.903 log_10_ (*p* = 0.4795) DPV 240. In contrast, calves in group 4 showed NA with an average titer of 1.505 log_10_ (*p* = 0.0369) on DPV 0. This titer progressively decreased, with a mean concentration of 0.401 log_10_ (*p* = 0.4795) on DPV 240 (Table 3). The gB-ELISA test revealed that group 1 showed positivity on DPV 15, and the same was detected up to DPV 240. In contrast, group 2 showed gB-positivity on DPV 0, which remained constant up to DPV 240. Furthermore, all animals showed negative gE-ELISA results during the entire vaccination period (Table 3).

On the day of the start of treatment with DMS (DPV 300), all calves were serologically negative for BoAHV-1. After cortisone administration, only calves in group 4 showed NA to BoAHV-1 with an average titer of 0.401 log_10_ (*p* = 0.3173) on DPT-DMS 10. This increased to 0.602 log_10_ (*p* = 0.0369) on DPT-DMS 120 (Table 4). The animals were positive for gB-ELISA on DPT-DMS 10, and the same result was detected up to DPT-DMS 120. In contrast, all animals after DMS treatment were negative in the gE-ELISA test throughout the experiment period (Table 4).

### 3.3. Virological Investigations

During the vaccination period, only the calves in group 2 released the vaccine virus after a booster on DPV 21, which lasted for 3 d (mean *Ct* = 33.00). After DMS treatment, none of the calves shed the virus during the experiment period. Throughout the experiments, virological investigations yielded negative results.

## 4. Discussion

In recent years, the European Commission has issued several regulations [4] and Delegated Acts [5,13] that allow Member States to submit control or eradication programs for different infectious diseases, including IBR. DIVA strategy has been applied for control programs where IBR is highly prevalent [3]. However, recently, the European study group on IBR, within the framework of the DISCONTOOLS’ [8], drafted a chapter on current gaps regarding IBR. Among these, the possibility of generating SNLC animals by administering a live attenuated *ts* vaccine in calves with passive immunity to IBR is described. SNLCs are economically important in BoAHV-1 regions where control programs are employed. In addition, failure to detect these animals could represent a potential risk for artificial insemination centers, genetic selection stations, and BoAHV-1-free herds or regions.

In this study, we evaluated the ability of two modified-live IBR marker vaccines (single and double deletions) administered to calves with passive immunity to generate SNLC animals.

After administration of the two modified-live IBR marker vaccines (A and B), no adverse reactions or clinical symptoms were observed in any animal. These results are in agreement with those of other studies, suggesting that the administration of single- or double-deletion IBR marker vaccines do not induce any adverse reactions in calves or water buffaloes [14,15,16,17,18,19]. However, it is known that the administration of the gE-/tk-deleted IBR live marker vaccines can lead to a short increase in rectal temperature of up to 2 °C, which was not observed in this study. Moreover, the results of this study contrasted those of a previous study that reported several adverse reactions in different herds of over 550 cattle after administration of a modified-live gE-deletion marker vaccine [20]. Bacilli et al. [21] also demonstrated that after the administration of a *ts* live strain of BoAHV-1, animals showed different adverse reactions, such as painful palpation, increased skin thickness at the inoculation site and increased rectal temperature from 6 to 168 h after the booster. The differences observed in adverse reactions between the results of our study and those shown in previous papers can be hypothesized to be due to a different type of vaccine administered to the cattle, age of the immunized animals, different types of adjuvants, and excipients mixed with the vaccine. Furthermore, the size of the group of vaccinated animals could also play an important role in evoking adverse reactions.

During the vaccination period, animals immunized with vaccine A did not shed the virus into the environment, except for those in group 2 (with passive immunity). All three calves shed the vaccine virus three days after the booster. The vaccine virus was detected using gB-specific real-time PCR; however, the viral isolation test yielded negative results. These differences in virus excretion could be because of the different sensitivities of the applied diagnostic tests [22]. Furthermore, the viral excretion observed in this study differs from those described in previous studies [23]. Strube et al. demonstrated the possibility of detecting the vaccine virus from nasal swabs collected from animals without passive immunity after intranasal administration of the vaccine [23]. Furthermore, Petrini et al. [24] revealed the absence of viral excretion in calves without passive immunity after vaccination with different live IBR marker products. The viral excretion observed in group 2 (with passive immunity) and compared to above -mentioned studies could be attributed to the mechanism of the immune evasion strategy of herpesviruses [25,26,27,28]. Furthermore, none of the animals immunized with vaccine B showed excretion of the vaccine virus. These results differ from those reported by Lemaire et al., who demonstrated that the administration of a live-attenuated *ts* vaccine resulted in high titer viral shedding over long periods of time in animals with passive immunity and in its absence [9].

Studies have employed the virus neutralization test using the WOAH protocol [12] and two commercial ELISA tests for both gB and gE antibodies of BoAHV-1 following the instructions and the manufacturer’s protocols. After immunization with the two different modified-live IBR marker vaccines, the calves with passive immunity (groups 2 and 4) progressively reduced their NA titers up to negative results on DPV 180 (group 2) and DPV 270 (group 4). These results were confirmed using gB-ELISA. On the day of the first vaccination, group 2 had a higher NA titer (2.308 log_10_) than group 4 (1.505 log_10_), and successively, these NA titers decreased progressively up to negative results on DPV 180 (group 2) and DPV 270 (group 4). Consistent with these findings, those of Lemaire et al. demonstrated that animals with passive immunity immunized with a live-attenuated *ts* vaccine became BoAHV-1 seronegative after an average period of 7 months [9]. In contrast, calves without passive immunity showed NA on PVD 15 (groups 1 and 3). Group 1 had a lower titer (0.401 log_10_) than that of group 3 (0.903 log_10_). Subsequently, group 1 reached the highest NA titer for PVD 90 (2.709 log_10_), whereas in group 3, the highest NA titer was observed on DPV 120 (2.208 log_10_). Thereafter, the NA titer progressively decreased until DPV 270 in both groups. These positive results were further confirmed using the gB-ELISA test.

The results obtained in groups 1 and 3 (absence of passive immunity) in terms of the humoral immune response were similar to those in other studies [9,28,29] and demonstrating their innocuity and efficacy. However, both modified-live IBR marker vaccines used in this study in groups 1 and 3 evoked a humoral immune response that lasted 240 days after the first immunization. This result contrasts with that described in other studies demonstrating a highly immunogenic immune response [30,31,32,33,34,35]. In addition, Romero et al. demonstrated that the administration of experimental gE-deleted IBR live marker vaccines inoculated intravenously induced long-term immunity, as the antibodies were detected up to 370 days after a single immunization [36]. During the vaccination period, investigations using the gE-ELISA test showed negative results, demonstrating that no BoAHV-1 field virus circulated in the experimental groups.

Concerning the latency induced by the modified-live IBR marker vaccines tested, the calves only in group 4 (immunized with vaccine B in the presence of passive immunity) generated SNLC. Moreover, when these calves were seronegative and treated with DMS, it was possible to reactivate the vaccine virus, showing only seroconversion to gB of both BoAHV-1 and NA. Humoral immunity occurs in the absence of viral shedding and clinical symptoms. The humoral immune response observed after reactivation of the latent vaccine virus was similar to that observed after primary infection. In particular, antibodies have been detected on DPT-DMS 10 [37]. These results differed from those reported by Lemaire et al., who demonstrated the generation of SNLC animals by immunizing calves with passive immunity with a live attenuated *ts* vaccine [9]. The study also reported the induction of SNLC in association with the excretion of the vaccine virus from 10^3.60^ to 10^5.70^ PFU/100 mg 3 to 7 days after DMS treatment. Moreover, during the reactivation of the vaccine virus, investigations using the gE-ELISA test always yielded negative results, demonstrating that no BoAHV-1 field virus was circulating in the experimental groups. This test is very important as it is used in several countries of the European Union as part of eradication programs in both serum and milk samples [3]. However, in this study, the reactivated vaccine was double-deleted (gE/tk negative), and in the international literature, it is contrastingly shown that the deletion of tk serves to avoid inducing viral latency [14,18,30,38]. Unfortunately, the pathogenic mechanism involved in viral reactivation after treatment with DMS in immunized calves in the presence of passive immunity is unknown. In contrast, no viral reactivation, clinical symptoms, or seroconversion were observed in groups 1, 2, and 3. Therefore, the results for the first three groups differed from those previously published [2].

The data obtained in this study did not support the hypothesis of the study concerning the use of the modified-live gE-deleted marker vaccine (single-deletion); however, for the first time, the results supported the research hypothesis that SNLC calves were generated after the administration of a gE-/tk-deleted IBR live marker vaccine in calves with passive immunity. To date, this phenomenon is unknown and is probably due to the activation of different cytokines that inhibit the humoral immune response [2].

The present study has some limitations. Cell-mediated immune responses were not investigated during the entire experimental period. Furthermore, only a small number of animals can be used because of the current European legislation on animal testing, which requires a reduced number of animals based on the 3 Rs principle [10]. However, the number of calves selected in this study was sufficient to obtain statistically significant results. A further limitation of this study is that we did not investigate vaccine viruses in the trigeminal ganglia. This survey was not conducted as it was not included in the experimental design.

Therefore, further studies are needed to evaluate the cell-mediated immune response in calves immunized with different modified-live IBR marker vaccines in the presence of passive immunity. Furthermore, it would be necessary at the end of the experiments to search for the vaccine virus from the trigeminal ganglia or develop new methods for detecting the BoAHV-1 genome from peripheral blood mononuclear cells and endogenous or microRNA from nasal secretion [39].

## 5. Conclusions

In conclusion, our results demonstrate that SNLC can be experimentally produced after immunization with a gE-/tk-deleted IBR live marker vaccine in calves with passive immunity. Moreover, after 5 days of intravenous DMS treatment, the virus was reactivated in the absence of both clinical symptoms and viral excretion. The results obtained in this study must be considered in European areas where IBR control/eradication programmes are in place, as SNLC animals are economically important. The failure to detect these animals may present a risk for artificial insemination centres, genetic selection stations and BoAHV-1-free herds or regions. Future studies are needed to investigate the cell-mediated immune response in calves immunized with different modified-live IBR marker vaccines in the presence of passive immunity. Finally, to confirm the data obtained in this study, future studies will be conducted on a larger number of animals, as well as to carry out viral latency studies.

## Figures and Tables

**Table 2:** Serological results obtained during the vaccination phase in groups 1 and 2.

Group	Test	DPV											
		0	15	30	60	90	120	150	180	210	240	270	300
	gE-ELISA ^a^	−	−	−	−	−	−	−	−	−	−	−	−
1	gB-ELISA ^b^	−	+	+	+	+	+	+	+	+	+	−	−
	NA ^c,d^	0.000	0.401	1.806	2.408	2.709	2.509	2.208	1.806	1.204	0.401	0.000	0.000
	gE-ELISA ^a^	−	−	−	−	−	−	−	−	−	−	−	−
2	gB-ELISA ^b^	+	+	+	+	+	+	+	−	−	−	−	−
	NA ^c,d^	2.308	2.107	2.107	1.706	1.706	1.003	0.702	0.000	0.000	0.000	0.000	0.000
	*p*-value	0.0039	0.0431	0.1213	0.1213	0.0431	0.0431	0.0463	0.0369	0.0369	0.0369	−	−

See Table 1 for vaccine identification; DPV, days post-vaccination; ^a^ gE-ELISA, IDEXX IBR gE Ab test, Maine, USA; ^b^ gB-ELISA, IDEXX IBR gB X3 Ab test, Maine, USA; ^c^ NA, neutralizing antibodies; ^d^ expressed as log_10_ of the reciprocal of the high dilution that inhibited cytopathology (mean value); *p*-value, indicates the statistically significant difference in NA titer between animals with and without passive immunity.

**Table 3:** Serological results obtained during the vaccination phase in groups 3 and 4.

Group	Test	DPV											
		0	15	30	60	90	120	150	180	210	240	270	300
	gE-ELISA ^a^	−	−	−	−	−	−	−	−	−	−	−	−
3	gB-ELISA ^b^	−	+	+	+	+	+	+	+	+	+	−	−
	NA ^c,d^	0.000	0.903	1.505	1.505	2.007	2.208	2.107	1.806	1.505	0.903	0.000	0.000
	gE-ELISA ^a^	−	−	−	−	−	−	−	−	−	−	−	−
4	gB-ELISA ^b^	+	+	+	+	+	+	+	+	+	+	−	−
	NA ^c,d^	1.505	1.505	1.505	1.505	1.505	1.405	1.204	0.602	0.502	0.401	0.000	0.000
	*p*-value	0.0369	0.6579	0.8166	0.8166	0.2463	0.0463	0.0369	0.0369	0.0369	0.4795	−	−

See Table 1 for vaccine identification; DPV, days post-vaccination; ^a^ gE-ELISA, IDEXX IBR gE Ab test, Maine, USA; ^b^ gB-ELISA, IDEXX IBR gB X3 Ab test, Maine, USA; ^c^ NA, neutralizing antibodies; ^d^ expressed as log_10_ of the reciprocal of the high dilution that inhibited cytopathology (mean value); *p*-value indicates the statistically significant difference in NA titer between animals with or without passive immunity.

**Table 4:** Serological results obtained during the reactivation phase in groups 3 and 4.

Group	Test	DPT-DMS											
		1	2	3	4	7	10	15	21	28	60	90	120
3	gE-ELISA ^a^	−	−	−	−	−	−	−	−	−	−	−	−
	gB-ELISA ^b^	−	−	−	−	−	−	−	−	−	−	−	−
	NA ^c,d^	0.000	0.000	0.000	0.000	0.000	0.000	0.000	0.000	0.000	0.000	0.000	0.000
4	gE-ELISA ^a^	−	−	−	−	−	−	−	−	−	−	−	−
	gB-ELISA ^b^	−	−	−	−	−	+	+	+	+	+	+	+
	NA ^c,d^	0.000	0.000	0.000	0.000	0.000	0.401	0.602	0.803	0.903	0.903	0.903	1.003
	*p*-value	−	−	−	−	−	0.3173	0.1138	0.1138	0.1213	0.1213	0.1213	0.0369

See Table 1 for vaccine identification; DPT-DMS, days post-dexamethasone (DMS) treatment; ^a^ gE-ELISA, IDEXX IBR gE Ab test, Maine, USA; ^b^ gB-ELISA, IDEXX IBR gB X3 Ab test, Maine, USA; ^c^ NA, neutralizing antibodies; ^d^ expressed as log_10_ of the high dilution that inhibited cytopathology (mean value); *p*-value indicates the statistically significant difference in NA titer between animals with or without passive immunity.

## Data Availability

Data are contained within the article.
